# News and Notes

**Published:** 1898-10

**Authors:** 


### *NEWS AND NOTES.*

Dr. E. W. Boyd has returned from a year's study of general medicine and surgery in London, and is now located at 128 South Pryor street.

The Atlanta Medical College and the Southern Medical College of Atlanta, Ga., have been consolidated under the name of The Atlanta College of Physicians and Surgeons. Dr. W. S. Kendrick is Dean of the school. We wish it abundant success, and there is much territory to which it is justly entitled. There is no reason why Atlanta should not be a great medical center, and many good reasons why it should be selected for the training of physicians intending to practice in our Southern country.—*Southern Clinic.*

The *Journal of the American Medical Association* reports that Dr. Benjamin F. Wright, of Youngs, Ga., died on August 10th.

Dr. Richard J. Nunn, of Savannah, has been elected Treasurer again of the American Electro-Therapeutic Association. This is an office which has been filled by Dr. Nunn for several years.

The monument to Charcot, the celebrated Parisian neurologist, will be formally unveiled in the Sallpêtrière in Paris on October 23d.

Professor Wm. H. Welch has resigned as Dean of the Johns Hopkins University, and Professor Osler will fill this position in the future.

DR. L. W. GLAZEBROOK, of Washington, D. C., has reported the death of a child four and a half years old due to the use of bromoform in whooping-cough.

---

A MONUMENT will be unveiled in October at Copenhagen in honor of Wilhelm Meyer, the one man who brought before the profession the pathological importance of the presence of adenoid vegetations in the nasopharynx.

---

DR. DUNCAN, of the Twenty-second Kansas Volunteers, who ghoulishly desecrated the grave of a Confederate soldier near Camp Alger, has been courtmartialed and sentenced to five years' imprisonment. We think the penalty was too light.

---

TEACHER: "I hear your mother has scarlet fever. You must not come to school till she is well, as you might get the disease and give it to the other children."

Tommy: "Oh, you needn't worry, teacher. She is my stepmother and has never yet given me anything."—*Fliegende Blaetter.*

---

A NEW medical school has opened in Kansas City, Mo., known as the Columbian Medical School. The *Medical Index* says: "We believe that with the addition of this school Kansas City outranks St. Louis and every other city in the world in the number of medical colleges, regular and irregular, in proportion to its population."

---

GOVERNOR FOSTER, of Louisiana, has appointed the following physicians to constitute the State Board of Health: Dr. Emund Souchon, New Orleans, President; Drs. Hampden S. Lewis and Chas. A. Gaudet, New Orleans; J. C. Egan, Shreveport; T. T. Tarleton, Grand Coteau; R. L. Randolph, Alexandria, and W. G. Owen, Whitecastle.

WOMEN HONORED.—An Italian woman, Signorina Esther Bonomi, has obtained at the University of Genoa the first doctorate in medicine granted to a female student, in modern times at least, in Italy. Another woman, named Dr. Katharina van Tusschenbroek, has been appointed to the professorship of gynecology in the University of Utrecht.

---

DR. R. C. M. PAGE, who died a few months ago in New York, and who was so well known at the New York Polyclinic as a teacher, left a very excellent medical library. This has been presented to the University of Virginia by Mrs. Page. Dr. Page was educated at this latter institution, and the name belongs to one of the oldest families in the Old Dominion.

---

"I AM glad to find you better," said John Hunter, the famous surgeon, to Foote, the equally famous actor, one morning. "You followed my prescription, of course?" "Indeed I did not, doctor," replied Sam, "for if I had done so I would have broken my neck." "Broken your neck!" exclaimed Hunter, in surprise. "Yes," said Foote, "for I threw your prescription out of a three-story window."

---

THREE deaths and thirty-two serious cases of illness were recently caused by the eating of ice-cream at a summer boarding-house at Greenfield, in Ulster county, New York. An investigation is in progress, but it has not yet been determined whether the trouble was caused by ptomaines or by poisonous matter in the lemon extract with which the ice-cream was flavored. It is stated that the extract was purchased from a strolling peddler.

---

DR. PRATT AND THE CHICAGO ACADEMY OF MEDICINE.—Dr. James G. Kiernan, the Secretary of the Chicago Academy of Medicine, announces over his signature that the recently issued catalogue of the Chicago College of "Osteopathy" contains the assertion that the "orificialist surgeon," E. H. Pratt, one of its faculty, is a member of the Chicago Academy of Medicine. Dr. Kiernan says that he has never been a member, nor is he eligible.—*Med. News.*

---

AMERICANS KILLED IN BATTLE.—The official records of the War Department, as far as completed September 6th, show that during the war with Spain there were 33 officers and 231 enlisted men of the army killed in battle, a total of 264. This number includes all the lives lost in battle in the Philippines as well as those in Cuba and Porto Rico. The percentage of officers killed is strikingly large, and is said to be unprecedented in the battles of the world.—*Med. News.*

---

THE DENVER MEETING OF THE AMERICAN MEDICAL ASSOCIATION.—The generous hospitality accorded the members of the Association and invited guests at the meeting in Denver will be long remembered by those who accepted it. It is gratifying to know that after all bills, including the subscription of $2,000 to the Rush Mountain fund, had been paid by the local committee of arrangements, something over $4,000 remained in the hands of the committee and was returned *pro rata* to the subscribers to the entertainment.—*Med. News.*

---

MORTALITY FROM HEAT.—The extreme heat of the last two days of August and first four days of September caused a considerable increase in the mortality of the city. On September 2d there were 21 deaths from the direct effects of the heat reported, and on September 3d no less than 64, by far the largest record of any one day during the present season ; 45 of the deaths occurred in Manhattan Borough, 17 in Brooklyn, and 2 in Queens. On Sunday, the 4th, there were almost as many fatalities—60—and the death-list would no doubt have been still greater if it had been a working day. One of the victims of sunstroke was a member of the Seventy-first Regiment, New York Volunteers, now on furlough, who had safely passed through the tropical campaign of Santiago. It must be understood that these reports represent only a part of the actual mortality from the heat, as they do not include any cases regularly attended by physicians, but only those coming under the notice of the police, when the patients actually died on the street or shortly after their removal to hospitals.—*Med. News, N. Y.*

---

*The Lancet* of July 16 prints an amusing example of the conversation that passed between the French statistical department, anxious to obtain definite information on certain matters from the Turkish provinces. The Pasha of Damascus returned his list with the following answers:

"Question: What is the death-rate per thousand in your principal city? Answer: In Damascus it is the will of Allah that all must die; some die old, some young.

"Question: What is the annual number of births? Answer: We don't know; only God alone can say.

"Question: Are the supplies of drinking-water sufficient and of good quality? Answer: From the remotest period no one in Damascus has ever died of thirst.

"Question: General remarks on the hygienic condition of your city. Answer: Since Allah sent us Mohammed his prophet to purge the world with fire and sword there has been a vast improvement. But there still remains much to do. Eeverywhere is opportunity to help and to reform. And now, my lamb of the West, cease your questioning, which can do no good either to you or to any one else. Man should not bother himself about matters which concern only God. Salem Aleikum!"

*The Lancet* adds: "It is clear that the doctrine held by the Christian that God helps those who help themselves finds no favor in the municipal government of Damascus."

DEATH IN CRAPE.—"Many a woman has been laid in her coffin by the wearing of crape," writes a physician. "It is a sin to do or wear anything that hurts the health, and therefore I think it positively sinful for women to wear mourning. Even plain black is not wholesome. It is astonishing that this custom has not been abolished, for women have grown very sensible in the matter of dress. It would have been abolished long ago were it not for the fact that woman cares more for what other people say than she does for herself. For example, if a woman's husband dies she would not dare to go without a long crape veil and a crape-trimmed gown, or the world would say she was glad to get rid of him; after wearing it a while she is not brave enough to leave it off, for she knows the same relentless world will say that she is looking for number two. Women claim that mourning is a protection. If one is really grief-stricken one's own feelings are sufficient protection against society, and for my part I believe that crape and other mourning habiliments are often directly responsible for bad complexion, bad eyes, bad digestion, and bad temper."—*Dietetic and Hygienic Gazette.*

---

THE UNSUNG HEROES.—Dewey and Bagley and Hobson and Schley and Blue and Neville and Shaw, the heroes of Guatanamo, Capron and Shafter, and all the long list of men who have distinguished themselves in this war, officers and men, are deserving of all the generous praise that has been bestowed upon them for the brave deeds they have done and for their unfaltering devotion to duty. Their example is of inestimable value for its influence upon their fellow-citizens, both in civil and military life. They are a source of inspiration which tends to a higher order of patriotism in all the walks of life and all the duties of the citizen. The patriotic uplift is discoverable to the most distant part of the country, and in the remotest hamlet. It manifests itself in greater love for the flag, greater pride in citizenship, greater fidelity to civic duties. War is a great character-maker. The heroes of the field and of the wave are its exemplars, and under the spell of hero-worship men grow better. All honor to the Deweys and Hobsons and the rest of them. Yet there is one class of heroes whose praises are seldom sung and whose patriotic sacrifices receive but slight recognition in the dispatches from the front. But they are there, exposed in greater or less degree to the fortunes of war, doing their duty without hope of acknowledgment, and performing their daily and nightly tasks with a devotion that knows no flagging, and without the spur and the inspiration of the cavalry charge, or the shriek and crash of the flying shells on board the man-of-war. The surgeon's duties on the field and in the hospital tent preclude the incentive of excitement, and are attended by none of the dramatic action of war. No matter what the conditions, whether for the time in comparative safety or when his scene of operations becomes the center of the hottest fire, as sometimes happens, he stands to his post, offering his own that he may save the life of another. While remembering the heroes of the saber and the gun, do not forget the heroes of the scalpel and the probe. They are never court-martialed for cowardice. And no wonder. Bravery in the face of danger is no new thing for them. The volunteer surgeon has not learned courage and ceased to be afraid because of any "baptism of fire." He has faced danger before. The faithful and skillful doctor is always in the thickest of the fight in the battle of life. He has learned all there is to be learned of bravery and self-sacrifice in the midst of the deadly epidemic, and in the panic of pestilence and plague he acquired the courage which makes a hero of him now. God bless the doctors! They are the salt of the earth. No class of men see so much of the suffering and misery of life; none do so much to relieve distress without hope of reward in this world. And the doctors at the front—Congress passes no resolution of thanks to them, and presidents and public do not applaud their fidelity to duty. Why, then, are they there? What glory awaits their efforts? Their only reward is the satisfaction of duty done; the approval of their own consciences that their skill and experience and their limit of endurance have been spent without hesitation in the service of their country and in behalf of their fellow men. Remember the men, give praise and honor to sacrifice and courage and steadfastness in the face of the enemy, and remember the unsung heroes too.—*Minneapolis Journal, July 14.*

THE following is the official program of the meeting of the Tri-State Medical Society of Alabama, Georgia, and Tennessee, which will be held in Birmingham, Ala., on October 25, 26, 27, 1898:

President's Address—J. A. Goggans, Alexander City, Ala.

Early Diagnosis of Cancer of the Uterus—Thos. S. Cullen, Baltimore, Md.

Acute Anterior Poliomyelitis—E. D. Bondurant, Mobile, Ala.

A Case of Complete Obstruction of the Common Bile Duct by a Floating Gall-stone. Operation. Immediate Recovery and Restoration to Perfect Health. A Short Study of Gall-stones in the Common Ducts—W. H. Hudson, LaFayette, Ala.

A Simple Operation for Hemorrhoids without Injections, Ligature, Clamp, Cautery, or Crushing—R. R. Kime, Atlanta, Ga.

Total Amputation of the Penis so that the Patient Can Urinate Normally—H. M. Hunter, Union Springs, Ala.

Functional Impotence—W. H. Mangum, Georgiana, Ala.

Extirpation of the Pancreas—H. Berlin, Chattanooga.

Two Cases of Surgery—S. W. Purifoy, Lowndesboro, Ala.

Fracture of the Spine. Presentation of Two Cases—B. G. Copeland, Birmingham.

The Treatment of Intestinal Obstruction and Constipation by Electric Injections—R. P. Johnson, Oak Park, Ill.

A Case of Dislocation of the Humerus with Fracture of the Surgical Neck—Geo. S. Brown, Birmingham.

Conservative Gynecology per Rational Medication—R. H. Hayes, Union Springs, Ala.

Some Interesting Cases of Laparotomy—C. Hamilton, Rome, Ga.

Ectopic Gestation—W. E. B. Davis, Birmingham.

A Few Remarks on the Treatment of Puerperal Fever, with Illustrative Cases—J. C. Johnson, Glen Allen, Ala.

Puerperal Septicemia—Robt. B. Stapleton, Dothan, Ala.

Supravaginal, viz., Vaginal Hysterectomy—Clement Ritter, Selma, Ala.

Recent Advances in the Treatment of Gonorrhea—F. Goodwin Dubose, Florence, Ala.

Modern Treatment of Corneal Opacities, with Report of Cases —M. L. Heffelfinger, Huntsville, Ala.

Surgical Treatment of Trachoma—S. Kirkpatrick, Selma, Ala.

Keratitis—A. A. Greene, Anniston, Ala.

Purulent Ophthalmia: A New Method of Treatment—Frank Trester Smith, Chattanooga.

Syphilis of the Nose—S. L. Ledbetter, Birmingham.

Fevers of Alabama—Charles McAlpine Watson, Florence, Ala.

Color Blindness—H. S. Persons, Montgomery, Ala.

Some of the Emergencies of the Lying-in State—David A. Morton, Boaz, Ala.

Some Fevers of St. Clair County, Ala.—Eugene P. Cason, Ragland, Ala.

Continued Malarial Fever of Southeast Alabama—Wm. R. Belcher, Daleville, Ala.

Some Malarial Manifestations—J. N. Pearson, Florence, Ala.

Typhoid Fever—E. Eugene Mitchell, Oneonta, Ala.

Typhoid Fever—E. A. Mathews, Clanton, Ala.

Typhoid Fever—J. D. Gibson, Birmingham.

Typhoid Fever—S. W. Welch, Alpine, Ala.

Typhoid Fever, Report of Cases—C. L. Guice, Harris, Ala.

Some Suggestions in the Treatment of Typhoid Fever—J. C. LeGrand, Birmingham.

Diphtheria—H. L. Appleton, Cedar Bluff, Ala.

Scarlet Fever and Its Sequelæ—John Thomas Chapman, Selma, Ala.

Smallpox—Frank Prince, Bessemer, Ala.

Lobar Pneumonia, with Treatment—J. U. Ray, Jr., Blocton, Ala

Chorea—S. W. Fain, Chattanooga.

Organic Disease of Mitral Valve—J. T. Seay, Fern Bank, Ala.

A Case of Addison's Disease Treated with Adrenal Extract—H. A. Moody, Bailey Springs, Ala.

Disobedience to the Mandates of Nature is Treachery of a Suicidal Tendency—P. G. Trent, Sr., Roanoke, Ala.

Suggestions in the Healing Art—E. T. Camp, Gadsden, Ala.

Animal Heat—W. S. Edwards, Gadsden, Ala.

Eye Affections in General Diseases—J. L. Minor, Memphis, Tenn.

Membranous Colitis—Louis Wm. Johnston, Tuskegee, Ala.

The Treatment of Typhoid Fever—Julius Jones, Rockfork, Ala.

Curettage of the Uterus—John G. Clay, Thompson's Station, Tenn.

Puerperal Eclampsia—Thos. F. Moore, Linwood, Ala.

Laparotomy for Gunshot Wound of the Abdomen; Report of Case, with Unique Features—R. E. Fort, Nashville, Tenn.

Peritonitis; Report of a Case—D. S. Middleton, Rising Fawn, Ga.

The Treatment of Burns on Modern Surgical Principles—C. B. Jackson, Horse Creek, Ala.

A Few Remarks on Bone Surgery—M. Goltman, Memphis, Tenn.

Neurasthenia and Its Treatment—Chas. E. J. Smith, Atlanta, Ga.

Abortion—Emmett K. Moon, Bridgeport, Ala.

Communal Hygiene—Ernest B. Sangree, Nashville, Tenn.

The Management of the Puerperal State—C. C. Jones, East Lake, Ala.

Perfection of the Aseptic Technique—W. F. Westmoreland, Atlanta, Ga.

Hypodermic Medication—E. P. Nicholson, Valley Head, Ala.

Syphilis of the Nervous System, with Report of Cases—W. J. Love, LaFayette, Ala.

Diphtheria—W. D. Travis, Covington, Ga.

Smallpox in Alabama—G. B. Wimberly, Reform, Ala.

A Rational Treatment for Typhoid Fever—Gaius R. Johnson, Marion Ala.

Typhoid Fever—G. Manning Ellis, Chattanooga.

Abnormal Cardiac Phenomena: Their Differentiation and Significance—R. M. Cunningham, Pratt City, Ala.

The Treatment of Affections of the Singing Voice—Richmond McKinney, Memphis, Tenn.

Malaria—W. H. Bell, Oxford, Ala.

Fever Indigenous to the Cumberland Plateau—N. L. French, Wartburg, Tenn.

